# Intracardiac Blood-Concretions

**Published:** 1863-08

**Authors:** J. F. Miner


ART. III.—*Intracardiac Blood-Concretions*—BY J. F. MINER, M. D.

It is not with the view of presenting any new theories that the subject of heart clot and its influence in some cases of sudden and unexpected death, is introduced to the attention of the readers of the Journal; but rather with a desire to explain, if possible, the causes which have in several instances in this vicinity produced death when it was least expected.—Coagulation of blood within the heart is not an uncommon occurrence, and may take place, just previous to, or simultaneously with death. It is said it may also take place after death, though the formation we have reference to, it is believed is never made after death. The *post mortem* clot is a soft, gelatinous coagulum, which does not merit the name which has been given it. Every physician accustomed to making dead house examinations, is familiar with these fibrinous concretions; their importance and the symptoms they produce may not be so well understood as their physical appearances and character. They may be suspected in some instances during life, but they are much more frequently observed after death, and generally but little importance is attached to them, upon the principle that they are formed during the death struggle, and after the circulation has nearly ceased. It has also been looked upon by some physicians, ignorant of its character and composition, as an organized growth, and denominated "Polypus of the Heart," a most satisfactory cause of death indeed. This is quite excusable, since in some instances the concretion is quite firmly attached to the endocardium, and cannot be separated without injury of this membrane; it is also said to be more or less distinctly vascular, according to the period of formation previous to death. The name, however, sufficiently indicates the incorrect views which were held concerning these formations, and shows these observers were very ignorant of common *post mortem* appearances, and of the literature of both pylypoid growths and intracardiac blood-concretions. It would be interesting to inquire in many cases where these concretions are found, what influence they exerted upon the circulation, and what agency, if any, they had in producing death. That they are often unsuspected during life, and found only upon examination after death, is sufficiently certain, while the causes and time of this formation together with the effects upon the circulation, the physical and general signs of its existence, and its agency in causing death, are much less apparent. What then are its causes or the conditions of the circulation which most favor its formation? Coagulation of the blood in the chambers of the heart is the accident of which we speak, the causes of this so far as they are known, and the symptoms by which it is recognized, are points of special interest.

The conditions necessary to the coagulation of blood while in the heart or vessels, would appear to be stagnation in the currrent—obstruction to the circulation, or unnatural composition of the blood itself, or perhaps both these conditions combined. Obstruction of the circulation may be produced by a great variety of organic changes in the heart or vessels, but so far as observed valvular disease of the heart is but rarely attended with blood concretions, nor are cases of enfeebled circulation from other causes, from general debilitating disease. Anæmia, however produced, either from protracted disease, repeated hæmorrhage, long, continued, exhausting discharges, or other causes, is not the condition of the system or blood which seems to favor what is called "heart-clot;" which is an expressive name, and quite appropriate when properly understood. More sudden impressions upon the circulation at a time of comparative health appears to be more often attended by blood concretions.

It is not consistent with the object of these remarks, to consider the numerous inquiries which suggest themselves in connection with this subject, or to attempt to throw any new light upon the diagnosis, prevention or cure of this disease. A few cases illustrative of this condition, or supposed to be illustrative of it might be instructive, and more fully convey the views of the writer. That we often observe heart clot upon *post mortem* examinations which had no agency in producing death, there can be no doubt. That it is sometimes the immediate cause of death is also not improbable, and to this point only is attention directed.

"J. G., aged 22 years, of usual good health, but having suffered from acute rheumatism upon several occasions, was subjected to anæsthesia by chloroform, for the purpose of removing the great toe. Chloroform was administered by an inexperienced student of medicine, and continued longer than was necessary. Respiration and the circulation were suspended so that when the operation was made it was observed that there was no hæmorrhage. This period of interruption was so long continued as to create alarm for the immediate safety of the patient, though the heart did not at any time cease entirely its action, at least did not so far as could be ascertained. The toe was amputated and stump dressed. The respiration commenced and the circulation was restored to a quite healthy standard. Consciousness was perfect, and the patient conversed rationally, but appeared strangely and looked anxiously. At length the breathing gradually became more hurried, the countenance livid, the pulse irregular, the heart sounds, mixed and tumultuous, and all the symptoms exceedingly singular and indicative of fatal termination. About thirty-six hours after the administration of the chloroform the patient died. Twenty-four hours after death *post mortem* examination revealed no cause of death. Brain not examined; heart natural; a straw-colored blood clot in the right side of the heart, probably formed in the last moments of life. Does chloroform produce death in that way? Please write and tell me what you think was the cause of death."

The above is a condensed history of a case furnished not long since by a medical friend, and with other cases coming to our knowledge has stimulated the hasty preparation of this article. One other instance of a similar character and further illustration will be unnecessary:

Mrs. ——, in labor with her third child. Pains severe and labor progressing; chloroform to complete anæsthesia; child born while mother was unconscious. For half an hour before completion of labor pains were feeble and inoperative. Not having forceps and parts being fully dilatable, traction was made upon the head with the fingers used in the manner of forceps, and by this means the child was assisted into the world. The uterus did not contract readily to expel the after-birth, and some assistance was afforded by traction upon the cord. During that time there was considerable hæmorrhage, and the patient at one time seemed to be dead, so complete was the syncope. After a little the patient rallied and appeared quite comfortable. The effects of the chloroform passed off, and everything looked favorably. She however became restless, anxious and delirious.—Pulse indistinct, feeble, and very frequent; respiration very difficult and rapid; and in about six hours after the termination of labor she died.—The friends of this woman, especially those of them who are opposed to, and prejudiced against chloroform, lay her death at its door; but before determining this point it would be necessary to ascertain what influence, if any, it had in producing the condition of suspended circulation and respiration. It is perhaps an entirely new view of the dangers of chloroform to regard it as liable to produce death by inducing blood clot during the periods of suspended respiration which so often occur under its influence; still, in connection with hæmorrhage, which is supposed also to favor this formation, it may have more influence than has usually been supposed. Whatever interrupts the circulation, or greatly enfeebles it, must certainly be regarded as more or less operative in producing such result.

Authors upon diseases of the heart describe with sufficient minuteness the physical signs and general symptoms of this condition, and yet it must be confessed that diagnosis is rarely positive, and rests upon the general history and circumstances of the case, rather than upon any well defined local or general condition. That cases do occur, where the diagnosis is positive, is quite possible, but it is more common that this condition of distress, dyspnoea and death, are attributed to other causes. These violent symptoms are produced only in cases of rapid formation of blood clot, since when it is a product of slow growth the heart remains tolerant of the foreign substance, and the circulation and respiration is comparatively little affected by it.

The intimation that some cases of unexpected and sudden death depend upon this cause, is made only as a suggestion, and not as a demonstrated reality. It is a rational and plausable cause of death in the two cases referred to and briefly described, and yet it is not quite certain that the clot found in the one case acted in any way to interfere with the circulation or shorten life; while the second case where there had been hæmorrhage, might have died from other causes, and no heart clot is known to have been present. It is conjectural only, and belongs to that large class, "cause of death unknown." The hæmorrhage would greatly favor the coagulation of the remaining blood; but perhaps with our present knowledge upon this point we are not justified in regarding coagulation as positively the cause of death. Other cases might have been introduced where much less doubt would exist as to the nature of the case, where blood clot was found after death sufficient to produce the most serious effects upon the circulation, but these instances are recent, and an attempt to indicate their nature has prompted these reflections upon this subject mainly with the hope of calling attention to this form of disease.

